# Dual Pathways of Online Social Support on Sleep Quality in University Freshmen: The Suppression Effect of Psychological Resilience and the Moderating Role of Digital Literacy

**DOI:** 10.3390/bs16040603

**Published:** 2026-04-17

**Authors:** Xiangying Meng, Shuidong Feng

**Affiliations:** School of Public Health, University of South China, Hengyang 421001, China; xymeng_0815@163.com

**Keywords:** online social support, sleep quality, psychological resilience, digital literacy

## Abstract

This study aimed to examine the impact of online social support on sleep quality and the potential roles of psychological resilience and digital literacy in this relationship. A cross-sectional survey was conducted with 606 university freshmen from a college in Hengyang City, Hunan Province, China, using cluster random sampling. Data were collected using the Online Social Support Questionnaire, Pittsburgh Sleep Quality Index (PSQI; note that higher PSQI scores indicate poorer sleep quality), Connor–Davidson Resilience Scale, and Domestic College Student Digital Literacy Assessment Scale. Statistical analysis was conducted using SPSS 27.0, with mediation effects tested via the PROCESS macro. The analytical results demonstrated that online social support showed a significant negative predictive effect on the sleep quality of university freshmen (β = −0.11, *p* < 0.01); psychological resilience exhibited a suppression effect (i.e., opposing direct and indirect effects) between online social support and sleep quality; and digital literacy moderated the first half of the mediation pathway (β = 0.18, *p* < 0.001). Collectively, this study shows that online social support directly harms sleep quality but indirectly benefits it by enhancing psychological resilience. Digital literacy serves as a key moderator that amplifies this beneficial indirect effect. These findings provide significant theoretical and practical insights for developing campus health promotion initiatives.

## 1. Introduction

### 1.1. Sleep Problems Among Adolescents

Sleep problems have become increasingly prevalent among young adults worldwide. According to the “2025 China Sleep Health Survey Report” released by the Chinese Sleep Research Society, 48.5% of adults experience sleep-related problems, indicating that sleep is gradually evolving into a public health concern. Previous studies showed a higher prevalence, with 40–44.2% of children and adolescents having sleep disorders (Jahrami et al., 2021; Díaz González-Colmenero et al., 2021). Sleep problems and sleep disorders can directly affect adolescents’ cognitive abilities (Mazza et al., 2024) and emotional regulation (Izabel et al., 2025). A growing body of research has documented the detrimental effects of sleep problems on adolescents’ academic performance. Yaghmour et al. (2023) demonstrated that sleep disturbances significantly predict lower grade point averages and increased academic difficulties among university students. College students with good sleep quality tend to demonstrate better academic adjustment and achieve higher academic performance (M. Wang et al., 2025).

For students who have just entered university, they are in a critical transitional period, simultaneously facing multifaceted challenges such as environmental adaptation, academic competition, interpersonal relationship restructuring, and future planning, which often renders their sleep quality vulnerable to disruption (Zhao & Zhang, 2024). Therefore, it is of significant theoretical and practical importance to thoroughly explore the protective and risk factors affecting the sleep quality of freshmen.

### 1.2. The Buffering Effect of Offline Social Support on Sleep

Prior to the digital age, research on social support and health focused almost exclusively on offline, face-to-face interactions. According to the social support buffering theory (Cohen & Wills, 1985), social support from family, peers, and other close relationships provides individuals with psychological and material resources that help them reappraise the threat posed by stressful events and cope more effectively, thereby mitigating the negative health consequences of stress. McLean et al. (2023) found that offline social support effectively buffers the negative impact of perceived stress on sleep quality, particularly during transitional periods such as the first year of university. Similarly, Singh et al. (2025) reported that psychosocial burdens can intensify the negative impact of poor sleep quality, while social support can help mitigate it. While offline social support is well-documented as a critical protective factor, its relevance to freshmen’s sleep quality is constrained by the unique context of university life: freshmen often face geographic distance from family. In this context, online social support has emerged as a complementary and even primary source of support for this population.

### 1.3. The Role of Online Social Support in Sleep Quality

Online social support is an extension of traditional social support forms within the digital environment, reflecting the degree of respect, acceptance, and understanding an individual gains during online social interactions; this construct typically encompasses four dimensions: instrumental support, informational support, belongingness support, and emotional support (Liang & Wei, 2008). Traditional research has primarily focused on the buffering effect of offline, real-world social support from sources such as family and peers on individual mental health. However, for the new generation of university students, the impact of online social support delivered via social media, online communities, and instant messaging tools on their mental health and sleep quality cannot be overlooked.

The effect of online social support on sleep quality may exhibits a “double-edged sword” characteristic: on one hand, the supportive interactions on social networking sites can reduce loneliness and social anxiety, benefit adolescents’ mental health (Z. Zhou & Cheng, 2022), and thereby improve sleep quality; on the other hand, online interactions may also trigger social comparison, cyberbullying, information overload, and “fear of missing out,” leading to increased anxiety and stress, which subsequently impairs sleep quality (Song et al., 2023). This paradoxical pattern is further complicated by evidence showing that groups with higher online social support are generally associated with better mental health, but also higher problematic internet use (Z. Zhou et al., 2025), suggesting that the very mechanisms that confer psychological benefits may simultaneously increase vulnerability to maladaptive online behaviors that disrupt sleep. This contradiction suggests the necessity of further clarifying the specific mechanisms and boundary conditions through which online social support affects sleep quality. Based on this, this study proposes:

**Hypothesis** **1.** *Online social support has a direct effect on the sleep quality of university freshmen.*

### 1.4. The Mediating Role of Psychological Resilience

From the perspective of resource transformation, social support, as an external resource, needs to be transformed into health outcomes (sleep quality) through an individual’s internal capabilities (psychological resilience). Psychological resilience refers to an individual’s ability to demonstrate positive adaptation and effective coping when encountering setbacks, stress, or adversity (Yu & Zhang, 2005), serving as a potential bridge from stress buffering to sleep optimization. According to the Conservation of Resources theory (Hobfoll, 1989), social support is a very important external protective factor for psychological resilience, helping individuals conserve psychological energy and enhance their ability to cope with stress. As a new form of social support, online social support may similarly promote mental health by strengthening their emotional regulation capabilities, self-efficacy, and positive cognitions (Knowles & Danzi, 2025). High levels of stress are a major predictor and contributing factor to poor sleep quality (Sanusi et al., 2022); therefore, psychological resilience, as an individual’s psychological resource for coping with stress, can mitigate the detrimental effects of negative emotions on sleep. Existing biological research has confirmed that psychological resilience can effectively modulate neuroendocrine fluctuations caused by sleep deprivation, plays a key role in this process, and ultimately promotes the improvement of individual sleep quality (Sun et al., 2014). Therefore, this study proposes:

**Hypothesis** **2.** *Psychological resilience plays a mediating role in the effect of online social support on sleep quality.*

### 1.5. The Moderating Role of Digital Literacy

The online environment is inherently complex and presents unique challenges not encountered in offline interactions. These include risks such as information overload, exposure to negative or misleading content, upward social comparison, cyberbullying, and addictive use patterns (M. Zhou & Zhi, 2023; Nor et al., 2025; Kasturiratna et al., 2025; Domoff et al., 2022). Given these challenges, the extent to which individuals benefit from online social support is unlikely to be uniform; this is likely dependent on their own level of digital literacy. Digital literacy is a comprehensive competency concept, encompassing the ability to critically use digital technologies, effectively access, understand, communicate, integrate, and create digital resources, along with possessing safety awareness and cultural literacy (Geng, 2020). The Digital Education Action Plan (2021–2027) highlights the promotion of high-performing digital education ecosystems and the enhancement of digital skills and transformation capabilities as strategic priorities for digital education (European Commission: Directorate-General for Education, Youth, Sport and Culture, 2023).

Digital literacy can be conceptualized as a moderator that shapes how online experiences translate into psychological outcomes. Individuals with high digital literacy are better equipped to filter beneficial information from noise, identify high-quality social support, and, to some extent, reduce negative social comparison and psychological risks (Vissenberg et al., 2022; Sechi et al., 2025). As a result, they are more likely to experience online social support as a resource that fosters psychological resilience. Conversely, individuals with insufficient digital literacy are more susceptible to interference from negative factors in the online environment (Hu & Qian, 2025), potentially turning what could be supportive into a stressor; based on the compensation effect model, those with low digital literacy may over-rely on nighttime social media for emotional venting as a substitute for more positive regulatory methods, thereby weakening the protective role of their own psychological resilience. Therefore, this study proposes:

**Hypothesis** **3.** *Digital literacy plays a moderating role between online social support and psychological resilience.*

**Figure 1:**
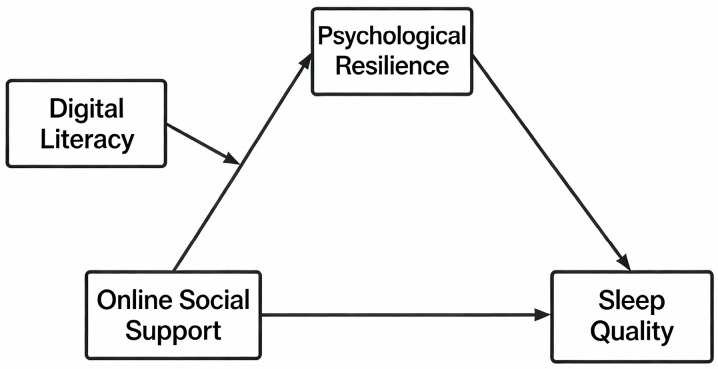
Hypothesis Model.

In summary, this study intends to construct a moderated mediation model (see Figure 1) to explore the pathway through which online social support affects sleep quality while also examining the mediating role of psychological resilience and the moderating effect of digital literacy.

## 2. Materials and Methods

### 2.1. Research Participants

This study adopted a cluster random sampling method, selecting freshmen from a university in Hengyang City, Hunan Province, China as the survey participants, with the class serving as the sampling unit. A total of 779 questionnaires were distributed. Exclusion criteria included: (a) diagnosed insomnia or other sleep disorders; (b) diagnosed anxiety or depressive disorders; (c) severe respiratory diseases; and (d) incomplete or invalid responses on the questionnaires. Based on these criteria, 606 valid questionnaires were obtained, yielding an effective response rate of 77.8%. The sample comprised 324 males (53.5%) and 282 females (46.5%); 46.9% were from urban areas, and 53.1% were from rural areas.

### 2.2. Measures

#### 2.2.1. Online Social Support

This study employed the Online Social Support Questionnaire developed by Liang and Wei (2008). It consists of 23 items forming four dimensions: peer support, information support, affection support, and instrumental support. Respondents rated each item on a 5-point Likert scale ranging from 1 (“strongly disagree”) to 5 (“strongly agree”). A higher total score indicates a higher perceived level of online social support. In this study, the Cronbach’s α coefficient for this questionnaire was 0.934.

#### 2.2.2. Sleep Quality

Sleep quality was measured using the Chinese version of the Pittsburgh Sleep Quality Index (PSQI), originally developed by Buysse et al. and revised by Liu and Tang (1996). This instrument assesses sleep quality over the preceding month. It contains 18 items, contributing to scores across seven components: subjective sleep quality, sleep latency, sleep duration, habitual sleep efficiency, sleep disturbances, use of sleeping medication, and daytime dysfunction. The component scores are summed to yield a global PSQI score, with higher scores indicating poorer sleep quality. Confirmatory factor analysis revealed that the factor loadings for sleep medication, sleep efficiency, and sleep duration were all below 0.30, which is consistent with previous research findings. Due to university students’ irregular sleep schedules, their engagement in various activities before falling asleep (such as exercising and using mobile phones), and the fact that Chinese university students rarely use medication to aid sleep, the factor loadings for these three components were relatively low (D. Zhang et al., 2021; Guo et al., 2014). It is important to note that all component removals were performed prior to computing the global score, not after. The global PSQI score in this study was calculated based solely on the remaining four components (subjective sleep quality, sleep latency, sleep disturbances, and daytime dysfunction). After removing these three components, the confirmatory factor analysis demonstrated good model fit: χ^2^/df = 2.641, GFI = 0.961, RMSEA = 0.052, CFI = 0.955, NFI = 0.930. In this study, the Cronbach’s α coefficient for the global PSQI score was 0.847.

#### 2.2.3. Psychological Resilience

Psychological resilience was evaluated using the Chinese adaptation by Yu and Zhang (2007) of the Connor–Davidson Resilience Scale (CD-RISC) originally developed by Connor and Davidson (2003). This 25-item scale measures resilience across three dimensions: tenacity, strength, and optimism. Items are rated on a 5-point Likert scale from 1 (“strongly disagree”) to 5 (“strongly agree”). Higher total scores reflect a greater level of psychological resilience. In the current sample, the scale demonstrated excellent internal consistency, with a Cronbach’s α coefficient of 0.951.

#### 2.2.4. Digital Literacy

Digital literacy was measured using the Domestic College Student Digital Literacy Assessment Scale (C. Fang & He, 2022), developed by the research group for the project “Study on Competence, Assessment System, and Development Strategies for Digital Literacy Education of University Students.” The scale includes 20 items covering five dimensions: information and data literacy, communication and collaboration, digital content creation, digital citizenship, and learning and development capabilities. Responses were recorded on a 5-point Likert scale from 1 (“strongly disagree”) to 5 (“strongly agree”), with higher cumulative scores indicating a higher level of digital literacy. Given that this study focuses on the mechanisms through which the processes of acquiring, filtering, and interacting with online social support impact sleep quality, we selected three theoretically relevant dimensions for measurement: information and data literacy, communication and collaboration, and digital citizenship. The remaining two dimensions—digital content creation and learning and development capabilities—were excluded because they showed weak and non-significant correlations with the core variables in our study (online social support, psychological resilience), as confirmed by preliminary correlation analyses (rs < 0.27, *p* > 0.06), whereas the three retained subscales showed moderate to strong correlations (rs ranging from 0.35 to 0.62, *p* < 0.01). After removing these two dimensions, the confirmatory factor analysis demonstrated good model fit: χ^2^/df = 2.031, GFI = 0.984, RMSEA = 0.041, CFI = 0.994, NFI = 0.988. The Cronbach’s α coefficient for the composite score of the three selected subscales was 0.924. The αcoefficients for the individual subscales were 0.873 for information and data literacy, 0.828 for communication and collaboration, and 0.872 for digital citizenship.

### 2.3. Statistical Analysis

Data preprocessing and analysis were performed using SPSS 27.0. Initially, descriptive statistics and bivariate correlation analyses were conducted on the core variables, and the reliability of all measurement tools was examined. Normality was assessed using Q-Q plots for each core variable. The data points fell approximately along the diagonal line for all variables, indicating an acceptable approximation of normality. Homoscedasticity was examined using Levene’s test, which yielded non-significant results for all variables across groups (all *p* > 0.05). Prior to analysis, variables were standardized into z-scores to facilitate comparison of effect sizes across variables. To test for mediation and moderated mediation effects, Hayes’ PROCESS macro (Version 4.1) was utilized (Hayes, 2009). Using the bootstrapping method with 5000 resamples, Model 4 was employed to test the simple mediation effect, and Model 7 was applied to examine the moderated mediation effect.

## 3. Results

### 3.1. Common Method Bias Test

Common method bias was evaluated using Harman’s single-factor test. The results indicated 11 factors with eigenvalues greater than 1, and the first factor accounted for 28.10% of the total variance, which is below the critical threshold of 40% (H. Zhou & Long, 2004). However, we acknowledge that Harman’s single-factor test has been widely criticized as a conservative and insufficient method for definitively ruling out common method bias (Podsakoff et al., 2012). This test is known to have low statistical power and may fail to detect bias when it exists. Therefore, while the result provides preliminary evidence that common method bias is not a major concern in this study, this evidence should be interpreted with caution.

### 3.2. Descriptive Statistics and Correlation Analysis

The results of the descriptive statistics and correlation analyses (see Table 1) indicate that online social support is significantly positively correlated with both psychological resilience and digital literacy; Sleep quality was significantly positively correlated with psychological resilience and digital literacy. Psychological resilience and digital literacy also demonstrated a significant positive correlation, and the correlation between online social support and sleep quality was not statistically significant.

### 3.3. Test of the Moderated Mediation Effect

Prior to examining the moderated mediation effect, a multicollinearity diagnostic was conducted on the variables, including online social support, psychological resilience, digital literacy, sleep quality, and gender. The Variance Inflation Factor (VIF) for each predictor was below 3, indicating no significant multicollinearity issues. Following the analytical procedure recommended by Wen and Ye (2014), we investigated the following pathways: the effect of online social support on sleep quality, the mediating role of psychological resilience in this relationship, and the moderating effect of digital literacy on the “online social support → psychological resilience” path. All continuous variables were standardized, and the analyses were performed using the PROCESS macro. It is important to note that higher PSQI scores indicate poorer sleep quality throughout this study.

First, the simple mediation effect was tested using Model 4 of PROCESS, with the results shown in Table 2 and Table 3. Equation (1) estimated the total effect of online social support on sleep quality without the mediator.

Y = 0.022 × X + 8.159(1)

Equation (2) tested the effect of online social support on psychological resilience.

M = 0.318 × X + 2.713(2)

Equation (3) included both online social support and psychological resilience as simultaneous predictors of sleep quality.

Y = −0.312 × X + 1.052 × M + 5.303(3)

Equation (1) revealed that the total effect of online social support on sleep quality was not significant (β = 0.01, t = 0.2, *p* > 0.05). J. Fang et al. (2012) noted that a significant total effect is not a prerequisite for mediation analysis. The analysis showed that online social support significantly and positively predicted psychological resilience (a = 0.318, β = 0.37, t = 9.92, *p* < 0.001). In turn, psychological resilience significantly and positively predicted sleep quality (b = 1.052, β = 0.33, t = 7.81, *p* < 0.001); this positive association indicates that higher psychological resilience was associated with better sleep quality (i.e., lower PSQI scores). The indirect effect was significant (ab = 0.334). It should be noted that the R^2^ value of 0.096 for Equation (3) is modest. From a practical standpoint, even a small amount of explained variance can be meaningful in the context of public health and intervention research. A 9.6% improvement in sleep quality, if achievable through targeted interventions, could translate into meaningful reductions in sleep disturbances at the population level. Notably, after controlling for psychological resilience, the direct effect of online social support on sleep quality was negative and significant (c′ = −0.31, β = −0.11, t = −2.73, *p* < 0.01). The opposing signs of the direct and indirect effects led to a suppression of the total effect. According to the criteria proposed by Wen and colleagues for distinguishing between mediation and suppression effects, the results indicate that psychological resilience has a suppressive effect in the relationship between online social support and sleep quality.

Subsequently, the moderated mediation effect was tested using Model 7 of PROCESS. As presented in Table 4, the interaction term between online social support and digital literacy significantly and positively predicted psychological resilience (β = 0.18, t = 6.21, *p* < 0.001). This indicates that the first stage of the mediation pathway (online social support → psychological resilience) was moderated by digital literacy.

**Figure 2:**
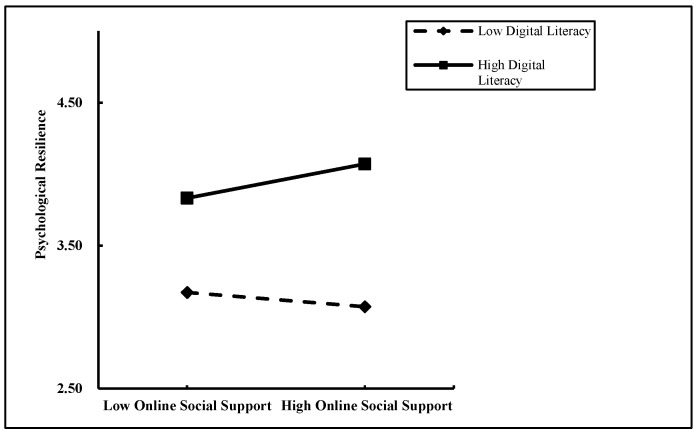
The Moderating Effect of Digital Literacy on the Relationship between Online Social Support and Psychological Resilience.

**Figure 3:**
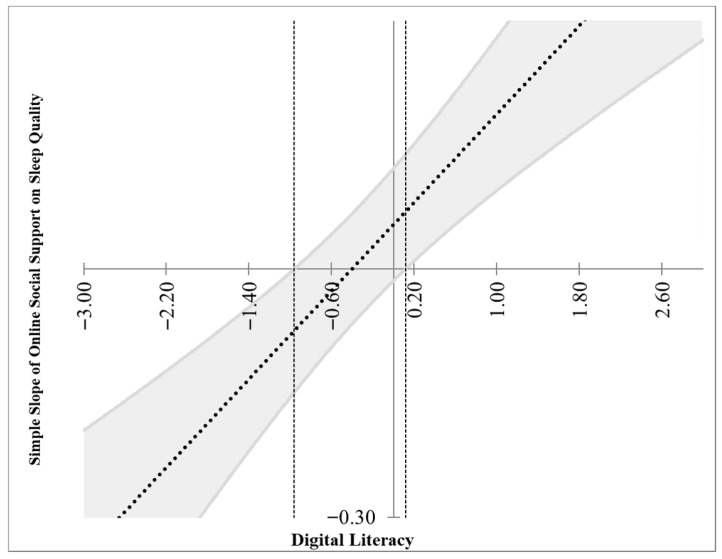
Johnson-Neyman plot illustrating the moderating effect of digital literacy on the relationship between online social support and psychological resilience. Note. The gray shaded area indicates the 95% CI for the simple slope. Vertical dashed lines denote Johnson-Neyman significance thresholds. Slopes are significant (*p* < 0.05) where the confidence interval excludes zero.

To further examine the pattern of this moderation, a simple slope analysis was conducted. Participants were divided into high and low digital literacy groups based on their scores. The relationship between online social support and psychological resilience was then analyzed within each group (see Figure 2 and Figure 3). For university freshmen with high digital literacy (M + 1 SD), online social support had a significant positive predictive effect on psychological resilience (β = 0.16, t = 5.09, *p* < 0.001). For those with low digital literacy (M − 1 SD), online social support showed a marginal negative predictive effect on psychological resilience, although this effect was small and only approached significance (β = −0.068, t = −1.93, *p* = 0.054). These findings demonstrate that the predictive effect of online social support on psychological resilience varies depending on the level of digital literacy, thereby confirming the moderating role of digital literacy between online social support and psychological resilience.

We next examined the conditional indirect effects of online social support on sleep quality via psychological resilience at different levels of digital literacy. As shown in Table 5, the indirect effect was negative and significant at low levels of digital literacy (−1 SD: effect = −0.071, 95% bootstrapped CI [−0.148, −0.003]), positive and significant at high levels of digital literacy (+1 SD: effect = 0.165, 95% bootstrapped CI [0.079, 0.247]), and non-significant at the mean level of digital literacy (effect = 0.047, 95% bootstrapped CI [−0.013, 0.104]). These results suggest that the indirect effect of online social support on sleep quality through psychological resilience may be moderated by the level of digital literacy: when digital literacy is low, the indirect effect is negative; when digital literacy is high, the indirect effect is positive.

## 4. Discussion

This study developed a moderated mediation model to examine the pathways and interrelationships among online social support, psychological resilience, digital literacy, and sleep quality in university freshmen. Contrary to the hypothesized simple mediation pathway, the data analysis revealed a more dynamic “competitive dual-path model” (MacKinnon et al., 2000). The influence of online social support on sleep quality is not linear; rather, it operates through two simultaneous and opposing pathways. Specifically, online social support was positively and indirectly associated with sleep quality through its relationship with psychological resilience, while simultaneously showing an independent direct negative association with sleep quality.

### 4.1. The Positive Mediating Pathway of Psychological Resilience

Consistent with the social support buffering theory (Cohen & Wills, 1985), the study found that online social support was significantly and positively associated with psychological resilience, which in turn was significantly and positively associated with sleep quality. This positive indirect effect partially validates Research Hypothesis 2 and aligns with the core tenets of the Conservation of Resources theory. For freshmen navigating the transition period, the emotional care, informational support, and sense of belonging derived from online communities constitute a significant psychosocial resource (Y. Zhang et al., 2023). This resource can serve a positive function when needed, mitigating the negative impact of external disruptions on sleep, fulfilling basic psychological needs (S. Wang et al., 2025), and thereby enhancing resilience in coping with various challenges in the new university environment. Individuals with higher levels of psychological resilience can effectively buffer the adverse effects of daily stressors on their sleep quality (Lei et al., 2023), and this trait has been confirmed to positively predict sleep quality (Arora et al., 2022). Yang et al. (2025) conducted two experimental studies and found a significant positive correlation between individuals’ cortisol awakening response (CAR) variability and their level of psychological resilience. Individuals with high psychological resilience demonstrated greater physiological regulatory flexibility and adaptability in their hypothalamic–pituitary–adrenal (HPA) axis when confronted with sleep disturbances. This effective physiological regulation serves as a crucial foundation for maintaining sleep homeostasis and facilitating the restoration of sleep quality. Consequently, psychological resilience may enhance individuals’ overall physiological and psychological adaptability in the context of online social environments, thereby indirectly exerting a protective effect on sleep quality. This pathway indicates that online social support can indeed serve as an important platform for freshmen to build psychological capital and facilitate adaptation, and its positive impact should not be overlooked.

### 4.2. The Direct Negative Association of Online Social Support

A more critical finding is that, after controlling for the mediating effect of psychological resilience, online social support showed a significant direct negative association with sleep quality. This clearly reveals the “double-edged sword” nature of online support, consistent with prior research reviewed in Section 1.3 (Z. Zhou et al., 2025). This direct negative impact may stem from several mechanisms independent of individual psychological resilience, although these mechanisms were not directly tested in our study. First, the time displacement and physiological arousal effect: frequent online socializing and content browsing can directly encroach upon sleep time, leading to delayed sleep onset (Cemei et al., 2024). Second, previous studies have shown that screen blue light exposure suppresses melatonin secretion (Rabiei et al., 2024), and emotionally charged online interactions may induce cognitive and physiological hyperarousal before sleep (Santiago et al., 2022). Third, upward social comparison pressure: browsing others’ updates can inadvertently trigger anxiety and directly impair sleep (S. Zhang & Liu, 2022). Although psychological resilience may buffer the immediate emotional impact of such content, the direct disruptive effect of stressful stimuli on sleep physiology persists. Fourth, potential delays in offline adaptation: according to Social Compensation theory, some freshmen may become overly reliant on or satisfied with online support, thereby reducing their proactive efforts to build real-world social connections. This can result in weak offline relationships and adversely affect sleep quality. This also underscores the importance of balancing online and offline life and enhancing digital literacy.

### 4.3. Theoretical Integration: The Competitive Dual-Path Model

**Figure 4:**
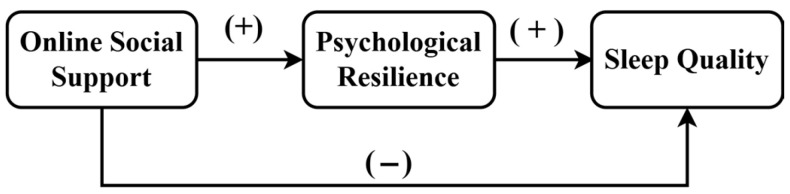
Competitive Dual-path Model.

By integrating the direct and mediating pathways, this study reveals a “competitive dual-path model” (see Figure 4). Here, psychological resilience does not function as a simple bridge but rather as a limited “resource filter.” It can transform positive elements of online support into personal psychological resources, thereby showing a positive indirect association with sleep. However, the positive effect of psychological resilience does not fully offset the direct negative association between online social support and sleep quality. As discussed in Section 2.3, the form of online interaction—such as exposure to social comparison, information overload, or potentially engaging with low-quality content—may act as an independent stressor. Consequently, psychological resilience, while capable of processing the supportive content, appears to have a limited buffering effect against the contextual challenges inherent in the online environment. This finding highlights the boundary conditions of psychological resilience’s role. When studying the relationship between online behavior and health, we must distinguish between the differential impacts of the content of behavior and the form of behavior.

### 4.4. The Protective Moderating Role of Digital Literacy

This study found that digital literacy significantly moderated the first stage of the pathway from online social support to psychological resilience, consistent with Hypothesis 3. Simple slope analysis showed, specifically, that freshmen with high digital literacy were more likely to show a positive association between online social support and psychological resilience, while those with low digital literacy showed a weaker or marginal negative association. This confirms our speculation that digital literacy is a crucial “skill of use.” Individuals with high digital literacy can more accurately discern information, seek high-quality supportive relationships, and effectively manage social boundaries, thereby enabling a more efficient conversion of online support into psychological resilience. This moderating effect indicates that cultivating digital literacy in the digital age is not merely a matter of technical education but a necessary strategy for promoting mental health.

### 4.5. Limitations and Future Research

While this study contributes new insights into the complex relationship between online social support and sleep quality by constructing a moderated mediation model, several limitations merit consideration and refinement in future research.

First, the geographic and demographic characteristics of the sample are relatively concentrated, being primarily drawn from freshmen at universities in a specific region of China. This limits the generalizability of the findings. Future studies should expand the sampling scope across diverse cultural contexts, institutional levels, and stages of student development to test the cross-group stability of the model.

Second, the cross-sectional design can only reveal associations among variables and cannot establish causality. Subsequent research should employ longitudinal tracking to dynamically examine the interplay of variables during the university adjustment period.

Third, the reliance on self-reported questionnaires may introduce common method bias and social desirability effects. Future studies could incorporate objective measures (such as wrist-worn sleep monitoring devices and digital platform usage logs) along with multi-source reporting to enhance data accuracy and ecological validity.

Fourth, although this study focused on key dimensions of digital literacy, it did not explore the potentially differential moderating pathways of its sub-dimensions (such as information literacy, communication literacy, and digital citizenship awareness). Subsequent research could conduct more granular analyses in this regard, thereby offering more specific guidance for designing targeted digital literacy education modules.

Fifth, several potential confounding variables were not controlled for. We did not measure total screen time or bedtime smartphone use, which may independently affect sleep quality. Regarding mental health, participants with diagnosed serious mental illnesses were excluded from the sample, but subclinical symptoms were not measured. Future research should include these variables to better isolate the unique effects of online social support content.

Finally, it should be noted that three components of the PSQI (sleep medication, sleep efficiency, and sleep duration) were removed due to low factor loadings in our sample, a finding consistent with previous research among Chinese university students (Guo et al., 2014; D. Zhang et al., 2021). Consequently, the modified PSQI global score was treated as a continuous variable rather than using the conventional >7 cutoff for classifying poor sleep. While this modification was empirically and contextually justified, it may limit the direct comparability of our findings with studies that employed the full PSQI. Future research should validate the modified four-component version of the PSQI in similar populations and establish population-specific cutoff scores if needed.

## 5. Conclusions

By examining the relationships among online social support, psychological resilience, digital literacy, and sleep quality in university freshmen, this study reveals the dual effects of online social support—it both promotes and impairs sleep quality. This finding underscores the complexity of online social support, indicating that it can serve as both a psychological resource and a health risk; thus, it should not be simplistically regarded as a protective factor. Psychological resilience does not function as a straightforward facilitative mediator in this relationship but rather exhibits a suppression effect. This suggests that while psychological resilience helps transform the positive aspects of online social support, it does not fully offset the direct disruptive effects of online usage behavior on sleep, highlighting the contextual boundaries of resilience within digital contexts. Digital literacy plays a critical protective moderating role in the model. This indicates that enhancing digital literacy is an entry point for improving university students’ adaptability to online environments and promoting their sleep health. In implementing health education initiatives, universities could consider incorporating digital literacy as a targeted intervention area to enhance freshmen’s competencies in information discernment, social media management, and other relevant skills.

## Figures and Tables

**Table 1:** Descriptive Statistics and Correlation Analysis Results for Each Variable (*n* = 606).

Variables	M ± SD	1	2	3	4
Online Social Support	3.25 ± 0.72	1			
Psychological Resilience	3.56 ± 0.63	0.378 **	1		
Digital Literacy	3.76 ± 0.65	0.464 **	0.679 **	1	
Sleep Quality	4.15 ± 2.27	0.012	0.289 **	0.197 **	1

Note. ** *p* < 0.01.

**Table 2:** Analysis of the Mediating Effect of Psychological Resilience.

Variables	Equation (1) (Dependent Variable: Sleep Quality)				Equation (2) (Dependent Variable: Psychological Resilience)				Equation (3) (Dependent Variable: Sleep Quality)			
	β	SE	t	95% CI	β	SE	t	95% CI	β	SE	t	95% CI
Gender	−0.063	0.17	−1.55	[−0.58, 0.069]	−0.089	0.048	−2.36	[−0.21, −0.02]	−0.034	0.16	−0.87	[−0.45, 0.17]
Online Social Support	0.008	0.11	0.2	[−0.20, 0.24]	0.37	0.032	9.92 ***	[0.25, 0.38]	−0.11	0.11	−2.73 **	[−0.54, −0.087]
Psychological Resilience									0.33	0.14	7.81 ***	[0.79, 1.132]
R^2^	0.004				0.15				0.096			
F	1.24				53.40 ***				21.23 ***			

Note. ** *p* < 0.01 *** *p* < 0.001.

**Table 3:** Mediation Effect Analysis.

Effect Type	Effect Size	Boot 95% CI		BootSE	t	Conclusion
		LLCI	ULCI			
Indirect Effect	0.334	0.082	0.163	0.021	15.901	Suppression Effect
Direct Effect	−0.312	−0.536	−0.087	0.114	−2.728	
Total Effect	0.022	−0.196	0.241	0.111	0.202	

**Table 4:** Test of the Moderated Mediation Effect.

Variables	Sleep Quality				Psychological Resilience			
	β	SE	t	95% CI	β	SE	t	95% CI
Gender	−0.14	0.16	−0.87	[−0.45, 0.17]	−0.08	0.04	−2.13 *	[−0.15, −0.01]
Online Social Support	−0.31	0.11	−2.73 **	[−0.54, −0.09]	−0.62	0.11	−5.45 ***	[−0.84, −0.40]
Digital Literacy					0.08	0.1	0.81	[−0.11, 0.26]
Online Social Support × Digital Literacy					0.18	0.03	6.21 ***	[0.12, 0.23]
Psychological Resilience	1.05	0.14	7.81 ***	[0.79, 1.32]				
R^2^			0.1				0.5	
F			21.23 ***				152.07 ***	

Note. * *p* < 0.05 ** *p* < 0.01 *** *p* < 0.001.

**Table 5:** Conditional Indirect Effect.

Digital Literacy	Effect	BootSE	BootLLCI	BootULCI
Low (−1 SD)	−0.071	0.036	−0.148	−0.003
Medium (Mean)	0.047	0.03	−0.013	0.104
High (+1 SD)	0.165	0.043	0.079	0.247

## Data Availability

Data is available upon reasonable request.
